# Role of Noradrenergic Inputs From Locus Coeruleus on Changes Induced on Axotomized Motoneurons by Physical Exercise

**DOI:** 10.3389/fncel.2019.00065

**Published:** 2019-02-26

**Authors:** Ariadna Arbat-Plana, Maria Puigdomenech, Xavier Navarro, Esther Udina

**Affiliations:** Department of Cell Biology, Physiology and Immunology, Institute of Neurosciences, Centro de Investigación Biomédica en Red sobre Enfermedades Neurodegenerativas, Universitat Autònoma de Barcelona, Bellaterra, Spain

**Keywords:** peripheral nerve injury, spinal circuitry, physical exercise, motoneuron, noradrenaline, locus coeruleus

## Abstract

Physical rehabilitation is one of the cornerstones for the treatment of lesions of the nervous system. After peripheral nerve injuries, activity dependent therapies promote trophic support for the paralyzed muscles, enhance axonal growth and also modulate the maladaptive plastic changes induced by the injury at the spinal level. We have previously demonstrated that an intensive protocol of treadmill running (TR) in rats reduces synaptic stripping on axotomized motoneurons, preserves their perineuronal nets (PNN) and attenuates microglia reactivity. However, it is not clear through which mechanisms exercise is exerting these effects. Here we aimed to evaluate if activation of the locus coeruleus (LC), the noradrenergic center in the brain stem, plays a role in these effects. Since LC is strongly activated during stressful situations, as during intensive exercise, we selectively destroyed the LC by administering the neurotoxin DPS-4 before injuring the sciatic nerve of adult rats. Animals without LC had increased microglia reactivity around injured motoneurons. In these animals, an increasing intensity protocol of TR was not able to prevent synaptic stripping on axotomized motoneurons and the reduction in the thickness of their PNN. In contrast, TR was still able to attenuate microglia reactivity in DSP-4 treated animals, thus indicating that the noradrenergic projections are important for some but not all the effects that exercise induces on the spinal cord after peripheral nerve injury. Moreover, animals subjected to treadmill training showed delayed muscle reinnervation, more evident if treated with DSP-4. However, we did not find differences in treated animals regarding the H/M amplitude ratio, which increased during the first stages of regeneration in all injured groups.

## Introduction

Peripheral nerve injury (PNI) results in a loss of motor, sensory and autonomic function in the denervated territory. Although peripheral axons have the ability to regenerate, this regenerative process is slow and functional recovery is often limited, mainly due to unspecific reinnervation of the target organs but also to alterations at the central level. The disconnection between the neurons and their target organs is accompanied by a disorganization of the central circuitry, in part due to the massive stripping of central synapses that axotomized motoneurons suffer. The most affected ones are the proprioceptive afferents from the muscle spindle that never recover basal values (Alvarez et al., 2010).

There is extensive literature on the use of activity-dependent therapies to improve axonal regeneration (van Meeteren et al., 1997; Al-majed et al., 2000; Sabatier et al., 2008; Asensio-Pinilla et al., 2009), and muscle reinnervation after sciatic nerve injury in rats (Asensio-Pinilla et al., 2009; Udina et al., 2011a). In general, when initiated during the denervation phase, moderate exercise training results in accelerated functional recovery (Gutmann and Jokoubek, 1963), whereas forced exercise or long-term hyperactivity tend to have a detrimental effect (Herbison et al., 1974; van Meeteren et al., 1997). Activity can also modulate the plastic changes observed after PNI, as neuropathic pain (Nam et al., 2001; Cobianchi et al., 2010) and hyperreflexia (Vivó et al., 2008; Asensio-Pinilla et al., 2009; Udina et al., 2011a). Increased activity, by reducing excitability of sensory neurons and synaptic stripping of motoneurons, attenuates maladaptive plastic changes in the spinal cord and central pathways (Mòdol et al., 2014; Arbat-Plana et al., 2015).

Although it is well-known that exercise exerts positive effects on the nervous system through modulation of brain derived neurotrophic factor (BDNF) (Hutchinson et al., 2004; Molteni et al., 2004; Gomez-Pinilla et al., 2012; Cobianchi et al., 2013), in a recent work we observed that pharmacological activation of TrkB, the specific receptor of BDNF, did not mimic all the effects of exercise on axotomized spinal motoneurons (Arbat-Plana et al., 2016). Moreover, we had previously shown that the integrity of sensory inputs from the injured limb is important for the maintenance of motoneuron perineuronal nets (PNN) induced by treadmill exercise (Arbat-Plana et al., 2015). PNN restrict plasticity and stabilize synapses (Kwok et al., 2011), and thus exercise, by activating proprioceptive and cutaneous receptors, facilitates preservation of central circuits after PNI. However, besides peripheral activation of muscles and sensory receptors, physical exercise is also activating a complex central circuitry related with locomotion and stress. Among it, the activation of noradrenergic neurons of the locus coeruleus (LC) may be important. Whereas ascending projections from this brain stem center, as part of the ascending reticular formation system, regulates arousal and attention (Berridge and Waterhouse, 2003), descending projections inhibit nociceptive transmission (Millan, 2002; Pertovaara, 2006) and modulate excitability of motoneurons (Heckman et al., 2003) in the spinal cord. In addition, noradrenaline release influences microglial function, by suppressing production of pro-inflammatory cytokines and promoting anti-inflammatory mediators (Heneka et al., 2010; Jardanhazi-Kurutz et al., 2011). Interestingly, an intensive protocol of treadmill exercise is able to attenuate the activation of microglia observed in the spinal cord after PNI (Cobianchi et al., 2010). This anti-inflammatory effect may be related to the increased activation of the LC induced by physical activity. However, the role of the LC in the activity-dependent-modulation of the spinal changes observed after axotomy has not been addressed yet. The LC can be chemically destroyed by using the neurotoxin N-(2-chloroethyl)-N-ethyl-2-bromobenzylamine (DSP-4), that selectively damages noradrenergic projections originating from the LC, inducing degeneration of noradrenergic terminals (Dooley et al., 1987; Dudley et al., 1990; Prieto and Giralt, 2001; Scullion et al., 2009). Therefore, we evaluated the role of the LC on the modulation of physical exercise on the plastic changes that motoneurons suffer after axotomy by submitting to TR control rats and rats that previously had suffered chemical ablation of the LC.

## Materials and Methods

### Experimental Design

Adult female Sprague Dawley rats (8 weeks old, 200–280 g) were housed with free access to food and water at room temperature of 22 ± 2°C under a 12:12-h light–dark cycle. All experimental procedures were approved by the ethics committee of Universitat Autònoma de Barcelona and followed the guidelines of the European Commission on Animal Care (EU Directive 2010/63/EU). For all surgical interventions, rats were anesthetized by intraperitoneal administration of ketamine (90 mg/kg, 0.9 ml/kg; Imalgen 2000) supplemented with xylazine (10 mg/kg, 0.5 ml/kg; Rompun 2%).

**FIGURE 1:**
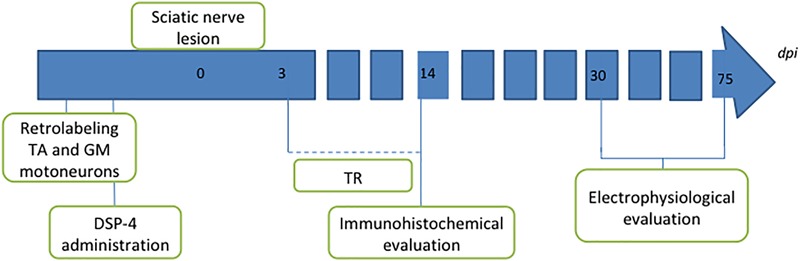
Diagram of the procedures performed in the study. Intramuscular injection of retrotracers was performed 1 week before the lesion to label motoneurons of both tibialis anterior (TA) and gastrocnemius medialis (GM) muscles. Two days later, DSP-4 was administered in the groups where LC had to be destroyed. Section and direct suture of the sciatic nerve was performed at point 0. Trained animals were exercised from 3 to 14 dpi. Animals were followed up for 14 days for immunohistochemical analysis or for 75 days for long-term electrophysiological assessment.

One group of animals was used as control group; these animals were only axotomized and did not receive any treatment (AX). Two groups of animals received DSP-4 before the surgery, as described below. Animals of one of these groups were subjected to exercise 3 days after the surgery (DSP-4+TR) whereas the other ones were not trained (DSP-4). A fourth group was subjected to the same protocol of exercise without receiving pharmacological treatment (TR). Each group was divided in two subgroups: one subgroup was euthanized 14 days after injury (short-term) to evaluate motoneurons changes and the other was euthanized 75 days after injury (long-term) in order to evaluate functional recovery (Figure 1 and Table 1). Contralateral side was used as control-intact side.

**Table 1:** Description of the different experimental groups used in this study, the type of follow-up and the number of animals used.

Group	Follow-up	Study
(1) Control group (AX)	Short-term	Changes around soma (*n* = 4)
	Long-term	EMG (*n* = 5)
(2) DSP-4 administration group (DSP-4)	Short-term	Changes around soma (*n* = 4)
	Long-term	EMG (*n* = 5)
(3) DSP-4 + Treadmill running (DSP-4+TR)	Short-term	Changes around soma (*n* = 4)
	Long-term	EMG (*n* = 5)
(4) Treadmill running (TR)	Short-term	Changes around soma (*n* = 4)
	Long-term	EMG (*n* = 5)

### Retrograde Labeling

Retrograde tracing [True Blue Chloride (TB, Setareh Biotech) and Fluorogold (FG, Fluorochrome)] were bilaterally applied 1 week before intervention to identify motoneuron pools of both tibialis anterior (TA) and gastrocnemius medialis (GM) muscles. A small incision to the skin was made to expose the muscle, and then two injections (2.5 μl/injection) were distributed within the muscle with a glass pipette using a Picospritzer (Arbat-Plana et al., 2015). The area of application was rinsed with saline to clean any remnants of the tracer and the skin wound was sutured.

### Surgical Procedure

Under anesthesia, the sciatic nerve was exposed at the mid-thigh and cut by using micro scissors. The proximal and distal stumps were rejoined with two epineural 10-0 sutures. Afterward, muscle and skin were sutured in layers, iodine povidone was applied to the wound, and the rats were allowed to recover in a warm environment under close observation.

### Noradrenergic Depletion

DSP-4 (Sigma Aldrich) was dissolved in sterile NaCl 0.9% saline and delivered as a single i.p. dose of 50 mg/kg according to the work of Lyons et al. (1989) within 10 min of preparation. DSP-4 was administrated 4 days before the injury to ensure LC destruction at the beginning of the treadmill protocol. Control animals received a single i.p. injection of saline solution. The doses of DSP-4 used were devoid of adverse effects, and therefore rats did not require special care during follow up.

### Treadmill Training Protocol

To acclimatize the animals subject to training exercise in a motor-driven rodent treadmill (Treadmill LE 8706, LETICA, Spain), they were placed on the treadmill for 60 min twice a week prior to surgery. During these training sessions, previous to surgery, shock grid intensity was set at 0.4 mA to provide a mild negative stimulus. The TR protocol was started 3 days after surgery and was carried out during 2 weeks. TR consisted of one session of TR 5 days/week with duration and intensity being progressively increased; running started at a locomotion speed of 10 cm/s that was increased 2 cm/s every 5 min, until a maximum speed of 30 cm/s for 60 min was reached during the final training session (Cobianchi et al., 2013).

### Immunohistochemical Analysis of Locus Coeruleus and Spinal Cord

Fourteen days after sciatic nerve injury, deeply anesthetized animals were transcardially perfused with 4% paraformaldehyde in PBS. Brain and L3–L6 spinal cord segment were removed, post-fixed for 4 h, cryoprotected in 30% sucrose, and stored at 4°C until use. To localize the LC in the brain, we used an acrylic array of coronal brain matrix (Acrylic brain matrices Alto, for small rat coronal, 175–300 gr). Both brain and spinal cord samples were embedded in Tissue-Tek, serially cut (25 and 20 μm thickness, respectively) with a cryostat, and collected onto gelatin-coated glass slides. All sections were first blocked with 10% normal bovine serum for 1 h, followed by overnight incubation at 4°C with anti-tyrosine hydroxylase (1:500, Sigma) for the brain sections or combinations of primary antibodies for the spinal cord sections: rabbit anti Synaptophysin (1:200, Covance), guinea pig anti VGlut1 (1:300, Millipore), guinea pig anti VGat (1:200, Synaptic Systems), Lectin from Wisteria Floribunda (1:100, Sigma), mouse anti GFAP (1:1000, Millipore), rabbit anti Iba1 (1:500, Wako). After washes, immunoreactive sites were revealed by species-specific secondary antibodies conjugated to 488 Alexa Fluor (1:200, Invitrogen), 538 Alexa Fluor (1:500, Invitrogen), Cy3 (1:200, Millipore), or Streptavidin 488 Alexa Fluor (1:200, Invitrogen). After 2 h incubation at room temperature, the sections were thoroughly washed, mounted on slides, and coverslipped with Fluoromount-G (SouthernBiotech). Back-labeled motoneurons were localized and images captured with a scanning confocal microscope (LSM 700 Axio Observer, Carl Zeiss 40×/1,3 Oil DIC M27). On the brain sections, LC was localized and images captured with an epifluorescence microscope (Nikon eclipse Ni DS-Ri2, 40×).

Image analysis, processing and regression analysis from motoneurons labeling quantification were performed by means of in-house software implemented in MATLAB R2012b (The Mathworks Inc., Natick, MA, United States). Firstly, back-labeled motoneurons were automatically selected and delineated and a constant threshold was used to segment and obtain an estimated average density for each labeling. As a control for the background, the software checked for each analyzed neuron that the soma had no labeling for the set threshold. Immunoreactivity was evaluated in a perimeter of 5 μm thickness surrounding the motoneuron soma (Arbat-Plana et al., 2015). Since some variability between animals, samples and processing can occur, similar amount of motoneurons from both sides were analyzed in each slide. For each animal, 10 to 15 motoneurons of each pool and each side were analyzed.

### Electrophysiology Tests

For the long term follow-up, motor reinnervation and H reflex were assessed by means of nerve conduction tests, performed at 30, 45, 60, and 75 days after surgery, using an electromyography apparatus (Synergy Medelec, Viasys HealthCare). Electrophysiological evaluation was performed under ketamine/xylazine anesthesia. During the test, the rat body temperature was kept constant between 34 and 36°C by means of a thermostated flat coil. The sciatic nerve was stimulated by two needle electrodes percutaneously inserted at the sciatic notch, applying single rectangular pulses of 0.1 ms duration up to the voltage required to obtain a maximal evoked response. The compound muscle action potentials (CMAP) were recorded from the TA, GM and plantar (PL) muscles with microneedle electrodes. Onset latency and amplitude from baseline to the maximal negative peak of the direct M wave and the reflex H wave were measured. The maximal H/M amplitude ratio was calculated for each muscle tested. The H wave (the electrophysiological equivalent of the stretch reflex) suffers a rate dependent depression (RDD) that is useful to corroborate that the recorded wave is truly the H wave. It is important to note that at early stages of regeneration, characterized by polyphasic CMAPs in the reinnervated muscles, the H wave can be masked. Thus, to ensure that the selected wave was the H wave, repeat stimulation was applied at increasing frequencies (0.3, 1, 3, 5, 10, 15, 20, and 30 pps) and at the threshold intensity for the H wave. The ratio between the amplitude of the last and the first H wave recorded for the different protocols was calculated, in order to evaluate the RDD. The different tests were performed also in the contralateral non-injured side to obtain control values for each animal.

### Statistical Analysis

For the immunohistochemical analysis, quantitative variables were normality assessed by Shapiro-Wilk test (Royston, 1993). For variables with a normal distribution one-way ANOVA and *post hoc* analysis by Bonferroni test were used to test the significance of the difference between the lesion side and the contralateral side. For non-normal variables such analysis was performed by Kruskall-Wallis test. SPSS 20.0 (SPSS Inc., Chicago, IL, United States) was used for statistical analyses. A nested design ANOVA test was used in order to determine if the variability was due to differences between the different motoneurons or between animals in each group. The H/M ratio and RDD data were analyzed by two way ANOVA with Bonferroni *post hoc* correction.

## Results

DSP-4 was administrated to destroy the LC and thus, to evaluate the effects of this center on the spinal changes mediated by TR during 14 days following PNI. Firstly, we explored the state of LC after DSP-4 administration using Tyrosine Hydroxylase antibody (TH), which is a useful marker for dopaminergic and noradrenergic neurons. To localize the LC we used sections 9.16 to 10.04 mm caudal to Bregma, following the Rat Brain Atlas in stereotaxic coordinates (Paxinos and Watson, 1998).

**FIGURE 2:**
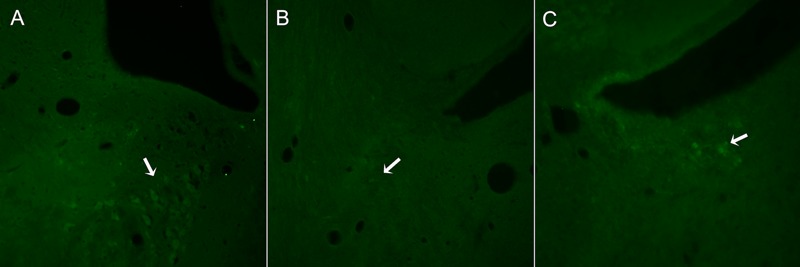
Representative images from sections of the brain stem regions where LC is localized in animals non-treated **(A)** or treated with DSP-4 **(B,C)** and immunostained against TH. The characteristic triangular shape of the LC nucleus **(A)** is lost 14 days after DSP-4 treatment. Hardly any TH+ neuron can be seen in the region (arrow, **B**), being some isolated neurons highly stained (arrow, **C**).

In control animals, a group of TH+ neurons organized in a typical triangular shape was easily localized in each side of the 4th ventricle (Figure 2A). In contrast, in DSP-4 treated animals, few TH+ neurons were localized (Figure 2B). In some animals, isolated brightly stained neurons were observed in the region (Figure 2C).

### Effects of DSP-4 Administration on Central Changes After Axotomy

**FIGURE 3:**
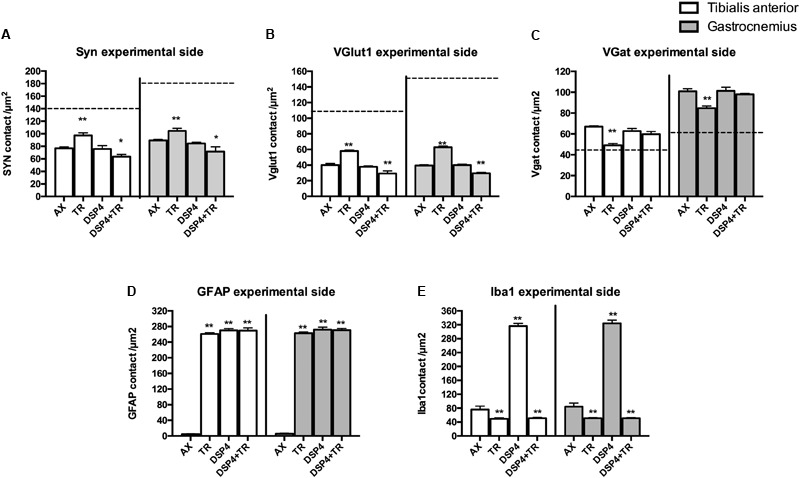
Quantitative analysis of synaptic stripping and excitatory/inhibitory synapses after axotomy in the different groups of animals. Evaluation of Synaptophysin **(A)**, VGlut1 **(B)**, VGat **(C)**, GFAP **(D)**, and Iba1 **(E)** in TA (white bars) and GM (gray bars) 15 days after sciatic nerve cut and suture in sedentary or trained animals, either treated with DSP-4 or untreated. Horizontal dotted lines indicate the average of motoneuron labeling in the non-injured side of both TA and GM motoneurons. Immunoreactivity for GFAP and Iba1 is so low in intact motoneurons that the horizontal dotted lines are missing in **D,E** graphs. Synaptophysin, Iba1 and GFAP values followed a normal distribution, whereas Vglut1 followed a non-normal one. Data are expressed as mean ± SEM, ^∗^*p* < 0.05, ^∗∗^*p* < 0.01, vs. AX group.

**FIGURE 4:**
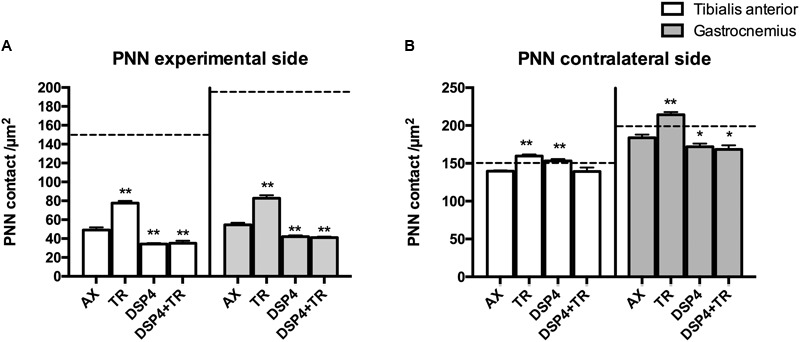
Quantitative analysis of perineuronal nets (PNN) 15 days after axotomy in animals subjected to treadmill or left untrained, either treated with DSP-4 or untreated, in both experimental side **(A)** and contralateral side **(B)**. Horizontal dotted lines indicate the mean of motoneuron labeling in the non-injured side of both TA and GM motoneurons. PNN values followed a non-normal distribution. Data are expressed as mean ± SEM, ^∗^*p* < 0.05, ^∗∗^*p* < 0.01, vs. AX group.

Synaptophysin (Syn) immunolabeling, a general marker for synapses, showed that the number of synaptic coverage of motoneurons was reduced after sciatic nerve injury, whereas TR partially preserved that loss (77 ± 3 Syn/μm^2^ and 97 ± 3 Syn/μm^2^ in AX animals vs. 89 ± 2 Syn/μm^2^ and 104 ± 4 Syn/μm^2^ in trained animals, in TA and GM, respectively, *p* < 0.01). No significant differences were found between animals treated with DSP-4 (83 ± 3 Syn/μm^2^ in both TA and GM) compared to control injured animals. DSP-4 administration combined with TR induced a marked decrease of synapses labeling on injured motoneurons compared to AX (63 ± 5 Syn/μm^2^ in TA and 71 ± 10 Syn/μm^2^ in GM, *p* < 0.05) (Figure 3A, 4).

Excitatory synapses were analyzed using VGlut1, a specific marker of proprioceptive afferents from the muscle spindle. Axotomized TA and GM motoneurons had a density of about 40 ± 2 per μm^2^, less than half than in intact motoneurons. After TR, the loss of excitatory synapses was also attenuated (60 ± 4 VGlut1/μm^2^ in both TA and GM, *p* < 0.01). Animals treated with DSP-4 did not show significant differences compared to injured control animals (42 ± 5 VGlu1/μm^2^). However, animals treated with DSP-4 subjected to TR showed a significant decrease of VGlut1 contacts on motoneurons in both TA and GM pools compared to AX and TR animals (30 ± 3 VGlut1/μm^2^, *p* < 0.01) (Figure 3B, 4).

The reduction of excitatory VGlut1 synapses suffered by axotomized motoneurons was accompanied by an increase of VGat labeled inhibitory synapses (67 ± 1 VGat/μm^2^ and 100 ± 3 VGat/μm^2^ in TA and GM, respectively). TR animals, showed a lower increase in inhibitory synapses after axotomy (49 ± 2 VGat/μm^2^ and 85 ± 2 VGat/μm^2^ in TA and GM, *p* < 0.01) than controls. In this case, no significant differences were found between DSP-4 and DSP-4+TR animals compared to control animals (Figure 3C, 4).

**FIGURE 5:**
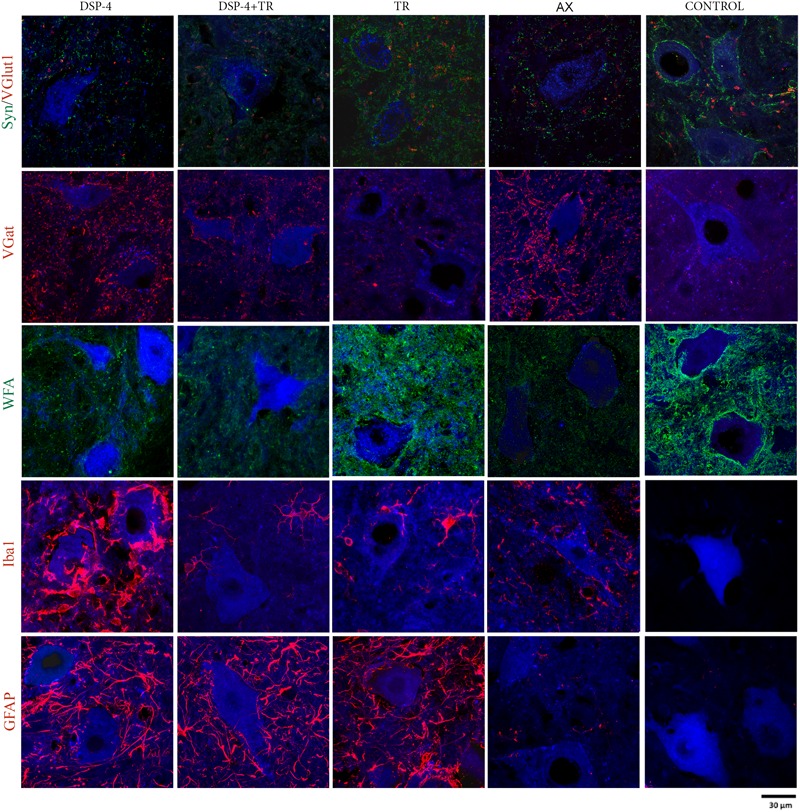
Evaluation of excitatory and inhibitory synapses, PNN, astroglia, and microglia reactivity surrounding axotomized motoneurons. All immunostainings were evaluated in confocal images of spinal cord regions containing back-labeled motoneurons (blue) of TA and GM muscles 15 days after nerve cut repaired with direct suture in the different groups of animals.

Wisteria floribunda immunoreactivity was used to analyze PNN. Axotomized motoneurons had a reduction in the amount of PNN (52 ± 5 PNN/μm^2^ in AX group compared to 150 ± 21 PNN/μm^2^ in intact rats) that was partially prevented if animals were subjected to exercise (75 ± 4 PNN/μm^2^; *p* < 0.01 vs. AX group). We observed that PNN immunoreactivity in injured motoneurons was lower in rats receiving DSP-4 (35 ± 1 PNN/μm^2^ and 42 ± 2 PNN/μm^2^ in TA and GM, respectively) than in only injured animals (*p* < 0.01). DSP-4+TR animals had also a low density of PNN surrounding motoneurons (35 ± 5 PNN/μm^2^ in both TA and GM). Interestingly, in DSP-4 group, GM motoneurons at the contralateral side showed a reduced PNN density (172 ± 8 PNN/μm^2^) compared to those of AX animals (182 ± 6 PNN/μm^2^), whereas this reduction was not observed in PNN from TA motoneurons (145 ± 18 PNN/μm^2^ in DSP-4 group and 139 ± 2 PNN/μm^2^ in AX group) (Figure 4, 5).

Astroglial reactivity, estimated by measuring the density of GFAP immunolabeling surrounding motoneurons, was significantly increased in animals subjected to TR (261 ± 1 and 263 ± 1 GFAP/μm^2^ in TA and GM, respectively) compared to the control injured group (5 ± 1 and 6 ± 1 GFAP/μm^2^, respectively, *p* < 0.001). Astroglial reactivity was also increased in both groups of animals treated with DSP-4 (around 271 ± 3 GFAP/μm^2^ for both muscles) similarly to TR only animals. Thus, administration of DSP-4 did not affect the modulation of astroglial reactivity induced by exercise (Figure 3, 5).

Iba1 immunostaining around axotomized motoneurons was used to evaluate microglial reactivity. Fourteen days after nerve injury, there was a marked increase of microglia labeling in control rats (76 ± 4 and 84 ± 5 Iba1/μm^2^ in TA and GM, respectively). DSP-4 administration significantly increased microglial reactivity (316 ± 4 and 324 ± 5 Iba1/μm^2^ in TA and GM, respectively, Figure 3) that also presented a phagocytic phenotype (Figure 5). Exercise drastically reduced microglia reactivity around injured motoneurons (49 ± 1 and 51 ± 1 Iba1/μm^2^ in TA and GM, respectively), and similar low activation of microglia was observed in animals treated with DSP-4 and also subjected to exercise (51 ± 1 Iba1/μm^2^ for both muscles), thus indicating that the modulation of microglia by exercise was not affected with the loss of LC.

### Muscle Reinnervation

Electrophysiological studies were performed to evaluate motor reinnervation during 75 days after sciatic nerve injury. The M wave values of the muscles at the contralateral side were similar in all animals. At the injured side, all the groups showed an increase in the amplitude of the M wave of the muscles tested along time, but with different progression.

**FIGURE 6:**
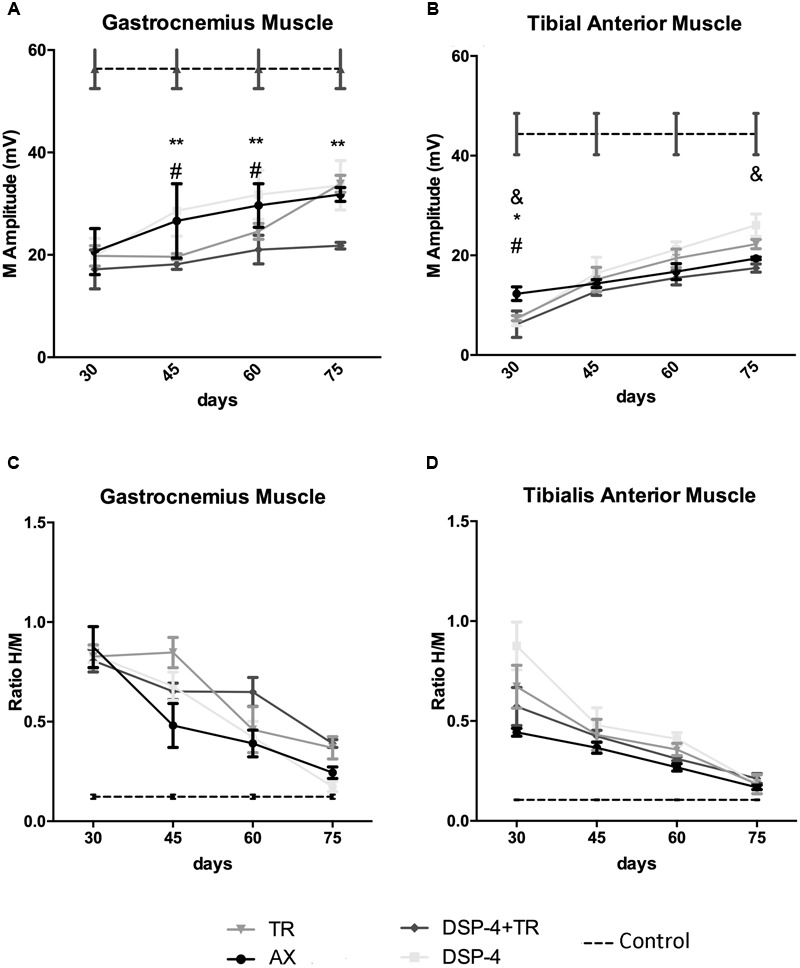
Plots of the amplitude of the CMAPs **(A,B)** and of the H/M ratio (ratio between the amplitude of the H reflex wave and the amplitude of the direct M wave after electrical stimulation of the nerve) **(C,D)** from the electrophysiological tests performed in the different experimental groups. Results recorded in GM **(A,C)** and TA **(B,D)** muscles at 30, 45, 60, and 75 dpi. Horizontal dotted lines indicate the average of CMAP or H/M ratio in the non-injured side of both TA and GM muscles. Data are expressed as mean ± SEM, ^∗^*p* < 0.05, ^∗∗^*p* < 0.01, ^∗^AX vs. DSP-4+TR; ^#^AX vs. TR; ^&^AX vs. DSP-4.

The groups of animals subjected to TR had lower M wave amplitude of the GM muscle at 45 and 60 days than the untreated group. However, at the end of follow up, the TR group achieved similar levels of reinnervation than the control, whereas the DSP-4+TR group remained with lower levels of reinnervation in both GM and TA muscles (Figure 6A,B). GM from DSP-4 group followed a similar progression of reinnervation than GM from control group. In contrast, at the end of follow-up, TA reinnervation in DSP-4 group was significantly higher than in AX group. Reinnervation of the PL muscles started in AX and DSP-4 groups at 45 dpi, and at 60 dpi in TR group. In contrast, in the DSP-4+TR group, we did not record M waves in the PL muscles at the end of follow up (data not shown), further indicating that motor regeneration was hampered by the combination of both treatments (Figure 6A,B).

Regarding the recovery of the H wave, there was an increase of the maximal *H/M* ratio after sciatic nerve injury, indicative of hyperreflexia, that followed a similar course to attain close to normal values at 75 days, without significant differences between groups (Figure 6C,D). In the injured side, the RDD rate was increased at all time points but again no differences between groups were observed.

## Discussion

The results of this study corroborate that a high intensity protocol of TR applied during 2 weeks after sciatic nerve injury was able to attenuate synaptic stripping and PNN loss around axotomized motoneurons, as we had shown previously (Arbat-Plana et al., 2015). In this work we aimed to analyze if the noradrenergic neurons of the LC play a role in these effects. Since a previous work pointed different responses to exercise in male and female mice (Liu et al., 2015), and we had previously evaluated the effects of two protocol of TR in female rats, here we used the more successful intensity of exercise to prevent central changes after PNI in female rats only.

The LC is strongly activated in stressful situations and besides its key role in arousal (Berridge and Waterhouse, 2003), it also plays an important role in modulating excitability of spinal motoneurons (Heckman et al., 2003) and nociceptive transmission (Millan, 2002; Pertovaara, 2006). It has recently demonstrated that contrasting modulation of pain-related behaviors is mediated with distinct noradrenergic neuronal populations, being the spinal-projections from the LC the ones related with analgesia (Hirschberg et al., 2017). Therefore, we disrupted the LC by administration of DSP-4 before performing a sciatic nerve injury in rats. DSP-4 is a neurotoxin that quite specifically destroys noradrenergic neurons of the LC, whereas respects similar neurons of other brain stem regions (Ross and Stenfors, 2015).

Destruction of LC did not affect synaptic stripping on axotomized spinal motoneurons. In contrast, when these animals were subjected to TR, the preventing effects of exercise on synaptic stripping were lost. In fact, they even showed more marked synaptic stripping and larger loss of VGlut1 synaptic contacts than injured control rats. Loss of noradrenergic projections from the brain stem also blocked the ability of TR exercise to revert the reduction in PNN. These results support the hypothesis that the increasing intensity TR protocol promotes activation of the LC, which play a role on the maintenance of synapses in axotomized motoneurons. As stated before, ascending projections from the LC influence arousal and attention (Berridge and Waterhouse, 2003) and optimize also task-performance (Aston-jones and Cohen, 2005). Therefore, loss of these ascending projections could indirectly affect spinal motoneurons. However, since descending neurons project directly onto the spinal cord, its logical to assume that are the descending neurons the ones mediating most of the effects we observed on DSP-4 treated animals. In fact, the LC is an important source of descending noradrenergic projections to the spinal cord (Grzanna and Fritschy, 1991), and thus its destruction would reduce the noradrenergic inputs that spinal motoneurons receive. Nevertheless, other brainstem noradrenergic nuclei, like A5 and A7, also send projections to the spinal cord. Since DSP-4 is a specific neurotoxin for LC neurons (Ross and Stenfors, 2015), we did not completely abolish all the NA projections that motoneurons receive and, therefore, stronger effects could be expected if all the spinal sources of NA had been ablated.

It is important to take into account that descending noradrenergic projections have a modulatory effect on the excitability of motoneurons, facilitating their activation when receiving other synaptic inputs (Heckman et al., 2008). Noradrenaline acts by facilitating persistent inward currents (PICs) in the dendrites (Hultborn et al., 2004; Perrier and Delgado-Lezama, 2005; Heckman et al., 2008, 2003). When activated, PICs amplify ionotropic synaptic inputs and are essential for normal repetitive firing (Lee and Heckman, 1999; Heckman et al., 2003), and thus for the production and facilitation of movement. Therefore, the loss of noradrenergic projections can reduce the effects of other synaptic inputs on motoneurons and limit the effects of increased activity induced by exercise. It is interesting to note that loss of noradrenergic projections also had effects on PNN of uninjured motoneurons, suggesting a widespread effect. After injury, animals that are not forced to run probably decrease their motor activity and thus, the loss of descendent noradrenergic projections does not potentiate the effects observed after the injury in motoneurons. In contrast, animals forced to run in a treadmill strongly activate these projections. It seems that in these situation, the lack of noradrenergic modulatory inputs in axotomized motoneurons is detrimental on the maintenance of the synaptic arbor and the PNN.

As we have already shown (Arbat-Plana et al., 2015), TR also affects the contralateral side, increasing the thickness of PNN around intact motoneurons, but it is unlikely that this increase has any relevant physiological effect in a context where plasticity is not triggered. In contrast, after a PNI, that lead to changes in the spinal circuitry, preservation of PNN could attenuate maladaptive plasticity and disorganization of the synaptic arbor of axotomized motoneurons, facilitating functional recovery.

Besides its ability to increase activity of the injured circuits and to preserve synapses and PNN in spinal motoneurons, TR exercise also modulates the inflammatory response observed in the spinal cord after PNI. Interestingly, noradrenaline has anti-inflammatory effects in the periphery (Kohm and Sanders, 1999; Sanders and Straub, 2002). Loss of the LC increased microglia reactivity around axotomized motoneurons in DSP-4 treated animals compared to controls. In contrast, animals forced to run in the treadmill downregulated microglia reactivity (Cobianchi et al., 2013). However, this anti-inflammatory effect of exercise at the spinal level seems independent of the LC noradrenergic system, since similar attenuation of microglia reactivity was observed in exercised animals after its depletion. Of course, NA from other sources could compensate the loss of noradrenergic inputs from LC and thus, modulate this inflammatory response.

Recent works have shown that the inflammatory response is important to switch the peripheral sensory neurons from a neurotransmitter state to a pro-regenerative state (Liberto et al., 2004; Frey et al., 2008; Niemi et al., 2013). Similarly, reactive microglia might influence the regenerative program in axotomized motoneurons. Since noradrenaline is a known anti-inflammatory (Heneka et al., 2010), its reduction in the spinal cord due to LC ablation, by increasing microglia reactivity around motoneurons might facilitate faster axonal regeneration and thus, explain the increased motor reinnervation observed in the DSP-4 group. On the contrary, TR exercise by itself attenuated the inflammatory response and delayed muscle reinnervation, as suggested by the lower amplitude of CMAPs observed in exercised animals during the first 8 weeks. Indeed, the effects of exercise on regeneration and muscle reinnervation are variable, depending upon intensity and duration of the protocol, and the period during which it is applied after the injury (Udina et al., 2011b; Gordon and English, 2015). It has been hypothesized that moderate exercise training enhances functional sensorimotor recovery, whereas forced intense exercise, such as the increasing intensity TR protocol applied here, may have a detrimental effect. In our study we also observed that at later stages, several weeks after stopping TR training, reinnervation improved. However, in exercised animals treated with DSP-4 this late increase of reinnervation was not observed, indicating that LC noradrenergic pathways are somehow mediating the effects of exercise on nerve regeneration.

It is interesting to note that that low intensity TR protocols, although being more effective promoting nerve regeneration and muscle reinnervation (Udina et al., 2011a), have limited effects on the preservation of synapses and PNN on axotomized motoneurons (Arbat-Plana et al., 2015), thus indicating that the protocol that facilitates regeneration differs from the one that favors maintenance of spinal circuitry.

Since the lack of recovery of the functional stretch reflex can be due to the permanent loss of proprioceptive afferent/VGlut1 synapses onto injured motoneurons even when they reinnervate the muscle (Alvarez et al., 2010; Rotterman et al., 2014) we also evaluated the state of the stretch reflex circuit by means of its electrophysiological equivalent, the H wave. The H/M amplitude ratio has been extensively used in the literature to measure spinal excitability. When the spinal reflex response is facilitated, the H/M ratio increases (Burke et al., 1999). At early stages of muscle reinnervation there is an electrophysiological hyperexcitability and the H/M ratio increases; as muscle reinnervation is consolidated, it returns to normal values (Valero-Cabré and Navarro, 2001). In our study we observed the same changes along time, without differences between experimental groups. However, it is argued that the electrophysiological recovery of the H reflex after PNI does not guarantee the functionality of the stretch reflex (Cope et al., 1994; Haftel et al., 2005; Bullinger et al., 2011).

In fact, we previously found that a low intensity protocol of TR (Vivó et al., 2008; Asensio-Pinilla et al., 2009; Udina et al., 2011a) attenuated such hyperexcitability by means of a decreased H/M ratio. However, this protocol was less effective preserving proprioceptive synapses on axotomized motoneurons than the high intensity TR protocol used here (Arbat-Plana et al., 2015). Therefore, evaluation of the functionality of stretch reflex is needed in order to fully understand the functional relevance of proprioceptive synaptic preservation by exercise after PNI.

In conclusion, LC seems to play a complex role on the effects of exercise on injured motoneurons. This center mediates the ability of physical exercise to activate motoneurons, and contribute to the maintenance of the normal synaptic inputs but does not participate in the anti-inflammatory effect of TR at the spinal level. Other sources of NA or either other monoamines like serotonin (Lopez-Alvarez et al., 2018) can also contribute to the effects of TR on the spinal cord. Interestingly, the sole elimination of LC in injured animals not subjected to exercise markedly increased microglia reactivity and improved early muscle reinnervation. In contrast, TR exercise, by reducing microglia reactivity, would delay motor regeneration and muscle reinnervation. However, this reduced microglia reactivity mediates other positive effects, such as attenuation of synaptic stripping and neuropathic pain (Cobianchi et al., 2010; López-Álvarez et al., 2015). Therefore, it seems that there is a delicate equilibrium between modulation of maladaptive plasticity and enhancement of regeneration. At this stage of research, exercise of moderate intensity seems more adequate than intensive protocols that can interfere with axonal regeneration. Use of combined protocols could also compensate the limitations of single therapeutic approaches.

## Author Contributions

AA-P contributed to the design of the experiments, performed all the experiments, analyzed the data, and wrote the manuscript. The work is part of her Ph.D. thesis dissertation. MP performed some of the experiments and collaborated in the writing of the manuscript. XN contributed to the design of the experiments and the writing of the manuscript. EU designed the experiments, performed some of the experiments, analyzed the data, and wrote the manuscript.

## Conflict of Interest Statement

The authors declare that the research was conducted in the absence of any commercial or financial relationships that could be construed as a potential conflict of interest.
